# Dietary Patterns, Bone Mineral Density, and Risk of Fractures: A Systematic Review and Meta-Analysis

**DOI:** 10.3390/nu10121922

**Published:** 2018-12-05

**Authors:** Edgar Denova-Gutiérrez, Lucía Méndez-Sánchez, Paloma Muñoz-Aguirre, Katherine L. Tucker, Patricia Clark

**Affiliations:** 1 Nutrition and Health Research Center, National Institute of Public Health, Cuernavaca 62100, Morelos, Mexico; 2 Clinical Epidemiology Research Unit, Hospital Infantil de México Federico Gómez, Ciudad de México 06720, Mexico; osteoclark@gmail.com; 3 Center for Population Health Research, National Institute of Public Health, Cuernavaca 62100, Morelos, Mexico; pmz.aguirre@gmail.com; 4 Department of Biomedical and Nutritional Sciences, University of Massachusetts Lowell, Lowell, MA 01854, USA; katherine_tucker@uml.edu; 5 Facultad de Medicina, Universidad Nacional Autónoma de México, Ciudad de México 06720, Mexico

**Keywords:** a posteriori, dietary patterns, bone mineral density, fracture risk, systematic review, meta-analysis, children and adolescent, young adults, adults and elderly

## Abstract

The aim of this systematic review was to assess the evidence on the relation between dietary patterns, bone mineral density (BMD), and risk of fracture in different age groups. Medline and Embase were searched for articles that identified dietary patterns and related these to BMD or risk of fracture through May 2018. Multivariable adjusted odds ratios (ORs) and 95% confidence intervals (95%CI) comparing the lowest and highest categories of dietary pattern were combined by using a random effects meta-analysis. In total, 31 studies were selected for review, including 18 cohorts, 1 case-control, and 12 cross-sectional studies, in the meta-analysis of Prudent/healthy and Western/unhealthy dietary pattern, BMD, and risk of fracture. There was evidence of a lower risk of fracture when intakes in the highest categories were compared with the lowest categories of Prudent/healthy dietary pattern (OR = 0.81; 95%CI: 0.69, 0.95; *p* = 0.01). In contrast, when intakes in the highest categories were compared with the lowest categories of Western/unhealthy dietary pattern, a greater risk of fracture (OR = 1.10; 95%CI: 1.02, 1.19; *p* = 0.01) was observed among men. The present systematic review and meta-analysis provides evidence of an inverse association between a Prudent/healthy dietary pattern and risk of low BMD and a positive relation between Western/unhealthy dietary pattern and risk of low BMD.

## 1. Introduction

Osteoporosis has been defined as a chronic disease characterized by low bone mass and bone tissue deterioration, with an increase in bone fragility and risk of osteoporotic fracture [1,2]. Due to its prevalence and contribution to morbidity, osteoporosis has been considered an important public health concern [3]. Osteoporotic fracture frequently results in disability, higher healthcare costs, and excess mortality [3,4]. Globally, osteoporosis-related fractures affect approximately 33% of women and 20% of men over the age of 50 years [5]. Low bone mineral density (BMD) has been considered to be a hallmark of osteoporosis, as well as a predictor of osteoporotic fracture [6,7,8]. Thus, a better understanding of the main prognostic factors of low BMD would have significant repercussions for public health.

Several risk factors for low BMD, such as genetic, endocrine, mechanical, and lifestyle (e.g., smoking status, alcohol consumption, physical activity, and calcium and vitamin D status) factors, have been established [9]. The evolution of low BMD to more serious conditions depends on how these risk factors could be modified throughout life. Existing research shows the important role of nutritional factors in the optimization of bone health [8]. Most attention has been given to the importance of calcium and vitamin D [10,11]. However, evidence is growing for the influence of other nutrients (sodium, magnesium, potassium, vitamin K, phosphorus, vitamin C, manganese, zinc, copper, and others) on bone health [12,13,14,15]. Additionally, studies have focused on foods and food groups [14,15,16,17,18], showing beneficial effects from fruits, vegetables, and whole grains as well as dairy products.

Although the association between diet, low BMD (osteopenia/osteoporosis), and fracture risk has been extensively evaluated, most studies considered only single factors, such as nutrients, foods, or food groups. More recently, to capture the synergies and cumulative effects of the overall diet and overcome problems of confounding by other factors of the diet, a dietary pattern analysis has been used to assess these relationships [19,20]. In general, two different approaches to extract dietary patterns have been defined: “a priori”, which concentrate on the construction of patterns or dietary indexes based on previous knowledge of a “healthy or unhealthy diet”; and “a posteriori”, which use exploratory statistical techniques (factor analysis, cluster analysis, or reduce rank regression analysis), and the observed dietary data [19,21,22,23]. As diets differ globally with respect to food choice and recommendations, we focus here on “a posteriori” methods because these statistical techniques could help us to avoid heterogeneity, and identify studies [24,25,26,27,28,29,30,31,32,33,34,35,36,37,38,39,40,41,42,43,44,45,46,47,48,49,50,51,52,53,54] that have evaluated the association between dietary patterns, BMD, bone mineral content (BMC), or fracture incidence. We conducted a systematic review with the objective of examining the evidence on the relationship between dietary patterns, BMD, BMC, and fracture risk and, when available, we analyzed this relation in different age groups. Furthermore, we conducted a meta-analysis to provide quantitative estimates of this association.

## 2. Material and Methods

### 2.1. Types of Studies

We included observational studies (cohort, case-control, and cross-sectional), reported as full-text, written in the English language, and published up to May 2018.

### 2.2. Types of Participants

Included studies focused on generally healthy people comparing BMD, BMC, or fracture with dietary patterns. The main outcome measures in these studies were: (1) BMD (Total or Lumbar spine or both); (2) BMC (Total); and (3) Risk of fracture. We define “healthy people” as those without a previous osteoporosis or osteopenia diagnosis and without chronic diseases, cardiovascular conditions, or autoimmune or inflammatory diseases (rheumatoid arthritis, osteoarthritis, fibromyalgia, multiple sclerosis, systemic lupus erythematosus, diabetes, or asthma). We excluded studies when participants had corticoid-steroid-induced or other secondary causes of osteoporosis.

Electronic strategic searches were designed with the following databases: MEDLINE by PubMed (1974 to May 2018) and EMBASE via Ovid (1993 to May 2018). The strategic search followed the PICOS framework, which acronym stands for: Population = open population; Intervention = dietary patterns; Comparison = other dietary pattern; Outcome = bone mineral density/bone mineral content/fracture; Study = observational (adapted to different digital libraries) with the limit: humans. Additionally, we evaluated other systematic reviews following the PICOS criteria according to our inclusion and exclusion criteria and considered the possibility of updating them. When we found different results, included articles were analyzed, compared with the PICOS from our review, and included in the evidence tables. We also reviewed the reference lists of all identified manuscripts for further identification of possible studies.

### 2.3. Data Collection and Analysis

#### 2.3.1. Selection of Studies

**Figure 1:**
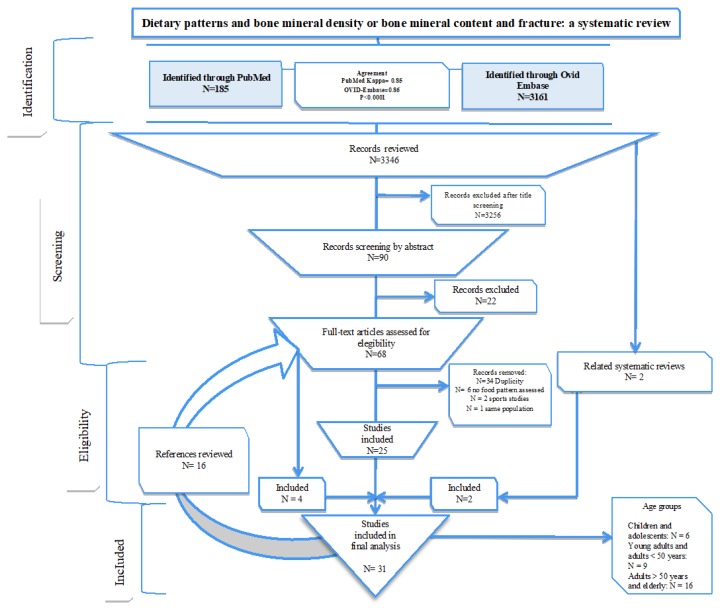
The systematic review flowchart.

Two reviewers independently screened titles and abstracts for all of the potentially relevant studies, and coded them as ‘retrieve’ (eligible or potentially eligible/unclear) or ‘do not retrieve’. Full-text study reports/publications were retrieved, and two reviewers screened them to identify studies for inclusion, or recording reasons for exclusion of those ineligible. Any disagreements were discussed and resolved and, if required, a third reviewer was consulted. Duplicates were excluded and multiple reports of the same study were collated so that each study (analysis) was unique in the review (Figure 1).

#### 2.3.2. Data Extraction and Management

One reviewer extracted study characteristics from included studies. A second reviewer spot-checked characteristics for accuracy against the trial report. For each study, we describe: methods (study design, study duration, details of any ‘run-in’ period, number of study centers and location (country), study setting, and date of study); participants (sample size, mean age, age range, sex, ethnicity, calcium and vitamin D intake (if available), and inclusion and exclusion criteria); and interventions (types of dietary patterns).

#### 2.3.3. Assessment of Potential Bias in Included Studies

Two reviewers independently assessed potential bias in each study using the Cochrane handbook and the criteria outlined in the *GRADEpro program* [55,56]. Disagreements were discussed and resolved and, if necessary, a third reviewer was consulted.

#### 2.3.4. Data Synthesis

We conducted a meta-analysis with available information from the identified studies with appropriate data.

## 3. Results

### 3.1. Results of the Search and Study Selection

From the literature search through the Medline-PubMed and Ovid-Embase databases, we identified and screened 3346 titles. Of these, we excluded 3256 because they did not conform to the PICOS criteria. To assess discrepancies between the reviewers, a concordance analysis was conducted; in general, “very good” strength of agreement was found (PubMed 0.85, *p* = 0.0001; and Ovid-Embase 0.86, *p* = 0.0001). After the first screening, abstracts of 90 articles were reviewed and, of these, a total of 68 papers were selected for full-text revision. Of the remaining 68 articles, the following were excluded: 34 studies for duplicity, 6 articles because they did not use an “a posteriori” method to assess the dietary patterns, 2 studies that focused on athletes, and 1 study that focused on the same population. Thus, a total of 25 studies were eligible for analysis. Subsequently, after a detailed review of the 25 articles, 18 new studies (including two related systematic reviews) were identified, their full texts were retrieved for detailed evaluation, and an additional 6 studies were included in the analysis. Finally, 31 studies were included in our systematic review (Figure 1). Of these, 12 studies, based on “a posteriori” methods to derive dietary patterns, were considered for the meta-analysis [27,28,29,34,36,39,42,44,49,50,52,53]. These 31 observational studies reported data on 175,060 participants. Six studies included information on children and adolescents; 9 included information on younger adults (<50 years); and 16 included information on older adults (≥50 years). Twenty-nine studies reported BMD or BMC measures using dual-energy X-ray absorptiometry (DXA). Only four studies reported fracture as an outcome and only in adults ≥50 years. For this clinical outcome, the population followed was 123,193 participants.

### 3.2. Included Studies

The methodological characteristics of the 31 studies [24,25,26,27,28,29,30,31,32,33,34,35,36,37,38,39,40,41,42,43,44,45,46,47,48,49,50,51,52,53,54] incorporated into the present analysis are reported in Table 1 and Table 2. These articles were published between 2002 and 2018 and included 18 cohort studies [24,25,26,30,31,32,33,39,40,41,42,43,44,45,46,47,52,53], 1 case-control study [54], and 12 cross-sectional studies [27,28,29,34,35,36,37,38,47,48,49,50,51]. Ten studies were conducted in Asia [27,28,29,36,38,43,44,49,50,54], 10 in Europe [24,25,31,37,39,40,41,45,46,52], 7 in North America [26,33,34,35,47,52,53], 3 in Australia [30,32,42], and 1 in South America [48]. Data from two studies [39,40] were from the Rotterdam Study.

### 3.3. Dietary Patterns Analysis for the Systematic Review

Twenty-three of the included studies derived dietary patterns with factor analysis methods, including principal components [24,27,28,29,31,32,33,34,36,37,38,40,42,43,44,45,48,49,50,51,52,53,54]; 4 reported results for dietary patterns defined by a cluster analysis (CA) [25,35,46,47]; and 4 used the reduced rank regression (RRR) approach [26,30,39,41].

Most of the studies that identified dietary patterns with factor analysis methods showed a significant association between BMD or BMC and one or two dietary patterns for all age groups considered. For example, in children and adolescents, significant and beneficial dietary patterns were primarily represented by high intakes of vegetables, fruits, low-fat milk and dairy products, whole grains, fish, beans, and nuts [24,27,28,29] as well as nutrients, such as calcium, contained in such foods [27]. These dietary patterns were labeled as “Dairy and whole grains” [24], “Calcium foods” [27], and “Milk and cereal” [28]. One study [27] identified an adverse dietary pattern, characterized by hamburgers and fried foods, pickles, snacks, cola beverages, coffee, and sugar, which was named a “Western” pattern. Among younger adults, one cohort study [32] and four cross-sectional studies [34,36,37,38] identified a positive association between BMD and dietary patterns distinguished by a high intake of vegetables, fruits, low-fat milk, dairy products, fish, legumes, whole grains, nuts, and olive oil [32,34,36,37,38]. These patterns were entitled: “Prudent” [34], “Dairy and fish” [34], “Fruit, milk, and whole grains” [36], and “Healthy” [38]. Additionally, four studies (two cohort and two cross-sectional studies) identified “Refined” [31,34] or “Western” [38] patterns, rich in red meat, fats, sugar and sweets, soft drinks, eggs, refined grains or cereals, and processed meats, as related to low BMD. Finally, in adults older than 50 years, four cohort studies [40,42,43,44] identified “Health conscious” [40], “Prudent” [42], “Milk and root vegetables” [43], and “Dairy” [44] dietary patterns, characterized by high intakes of fruits, vegetables, low-fat dairy, fish, legumes, high-fiber bread, water, and poultry [40,42,43,44], which were associated with a lower risk of low BMD or osteoporosis; while adherence to a “Western” [42,44], “Processed” [40], or “Refined cereal grains” [43] dietary pattern was associated with a higher risk of low BMD or osteoporosis.

Four studies used RRR to derive dietary patterns [26,30,39,41]; one was conducted in children and adolescents [26], another in young adults [30], and two in older adults [39,41]. In each of these, two dietary patterns were extracted; one was mainly characterized by high intakes of refined grains and cereals, red meats, processed meats, eggs, fats, sweetened beverages, and sweets. Only one study [26] reported a negative association between the first dietary pattern and BMC. The other three studies [30,39,41] did not find any significant association. Conversely, in all articles, a dietary pattern that was mainly represented by low-fat milk, fruit, dairy products, vegetables, fish, legumes, and whole grains was significantly related with greater BMD and/or BMC [26,30,39,41].

Four cohort studies derived a dietary pattern by means of a cluster analysis (CA) [25,35,46,47]. Of these, one was conducted in children and adolescents [25], one in young adults [35], and two in adults older than 50 years [46,47]. In general, these showed that individuals consuming a “Healthy” dietary pattern had higher BMD than those with an “Unhealthy” or a “Western” pattern. For example, in older adults, a diet high in fruits, vegetables, and breakfast cereal and limited in less nutrient-dense foods was associated with the highest BMD, particularly in men [47].

Only four studies, including three cohort studies [39,52,53] and one case-control study [54], reported associations between dietary patterns and risk of fracture. They were conducted in men and women over 50 years. One used RRR [39] and the others [52,53,54] employed factor analysis methods to construct dietary patterns. One study using a factor analysis [53] did not find any association between the “Prudent” or “Western” pattern and fracture risk among older men or women. The remaining three studies [39,53,54] identified significant associations between favorable dietary patterns (generally characterized by high intakes of vegetables, fruits, fish, lean poultry, legumes, nuts, whole grains, and water and labeled “Healthy” or “Prudent”) were significantly associated with a lower risk of fracture [39,53,54]. In contrast, the unfavorable dietary patterns [39,53,54] (rich in red meat, processed meat, poultry with skin, animal organ meat, cooking oil, soft drinks, hamburgers, hotdog, ice cream, doughnuts, margarine, and butter, and labeled “Sweets, animal fat” [39], “Western” (53), or “Energy-dense”), were associated with a higher risk of fracture [39,53,54].

### 3.4. Dietary Patterns for the Meta-Analysis

In total, 12 studies (4 cohort, 1 case-control, and 7 cross-sectional) were included in the meta-analysis of “Prudent/Healthy” and “Western/Unhealthy” dietary patterns, BMD, and risk of fracture.

Of these, nine studies (three cross-sectional in children and adolescents, two cross-sectional in younger adults, and five (two cohorts and two cross-sectional studies) in older adults), analyzed the association between a “Prudent/Healthy” dietary pattern and BMD. Four studies (three cohort studies and one case-control) evaluated the relation between risk of fracture and “Prudent/Healthy” dietary pattern among older adults.

The association between “Prudent/Healthy” and “Western/Unhealthy” dietary patterns and BMD or fracture was estimated using a random-effects meta-analysis with 95% CI.

### 3.5. Prudent/Healthy Dietary Pattern and BMD

In children and adolescents, a comparison of the highest to lowest category of the “Prudent/Healthy” dietary pattern in cross-sectional studies [27,28,29] resulted in an inverse association with low BMD (odds ratio (OR) = 0.49; 95% CI: 0.38, 0.63; *p* < 0.001). These studies showed no evidence of heterogeneity (*p* = 0.35, *I*^2^ = 4%). In two cross-sectional studies in adults younger than 50 years, a comparison of intakes in the highest quintile or quartile with the lowest category of the “Prudent/Healthy” dietary pattern [34,36] showed a significant reduction in risk of low BMD (OR = 0.52; 95% CI: 0.34, 0.80; *p* <0.01), with no evidence of heterogeneity (*p* = 0.09, *I*^2^ = 58%). In older adults, a comparison of the highest to the lowest category of the “Prudent/Healthy” dietary pattern in cohort studies [42,44] showed a pooled RR for low BMD of 0.58 (95% CI: 0.43, 0.78; *p* <0.001), with no evidence of heterogeneity (*p* = 0.54, *I*^2^ = 0%). A similar association was found with cross-sectional studies [34,49,50], with a pooled OR of 0.61 (95% CI: 0.41, 0.89; *p* = 0.01), and no evidence of heterogeneity (*p* = 0.48, *I*^2^ = 0%).

#### Prudent/Healthy Dietary Pattern and Risk of Fracture

**Figure 2:**
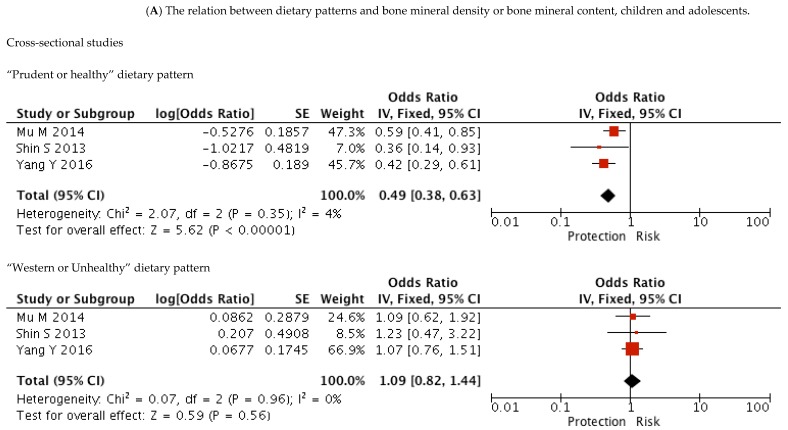

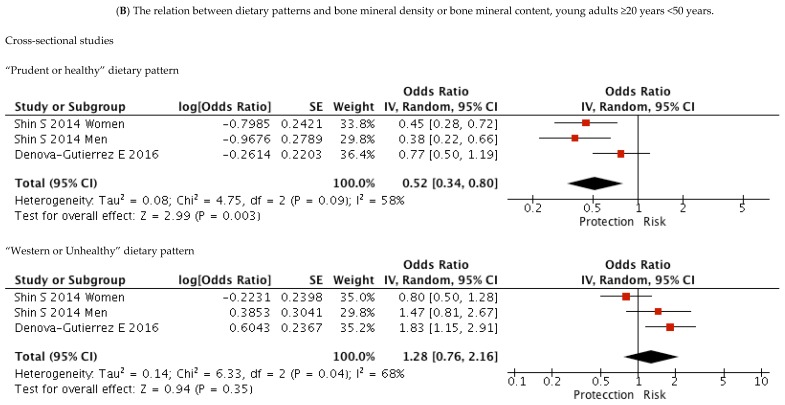

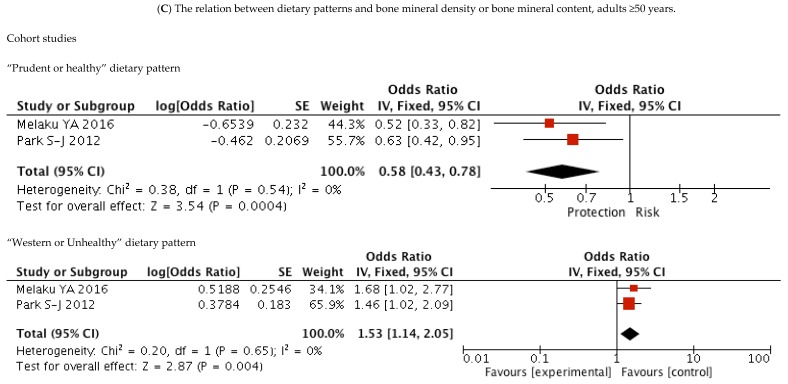

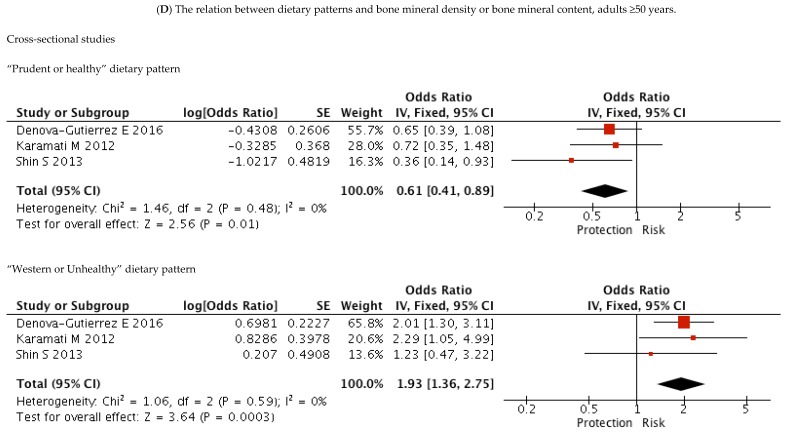

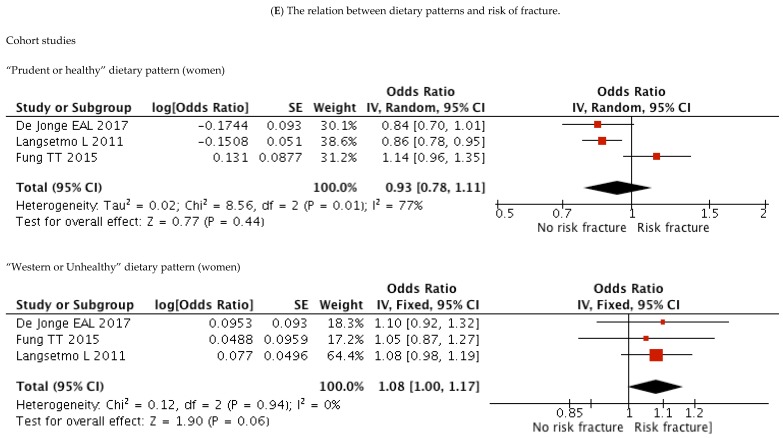

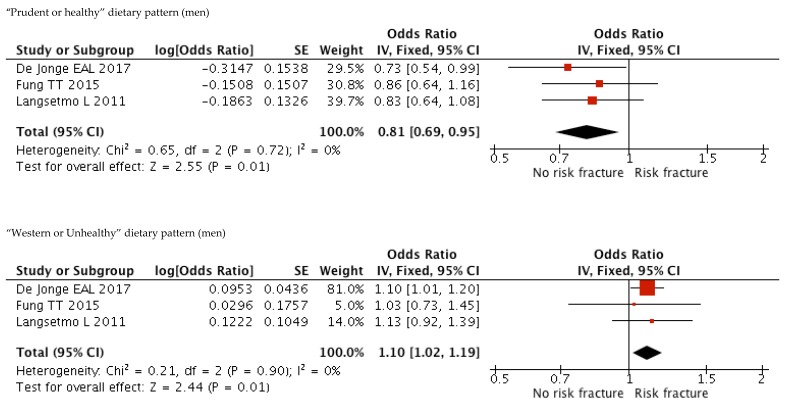
The dietary patterns (DPs) that were included in the meta-analysis stratified by “Prudent or Western” dietary pattern and type of study: (**A**) The relation between DP and BMD or BMC, children and adolescents; (**B**) The relation between DP and BMD or BMC, young adults >20 years <50 years; (**C**) The relation between DP and BMD or BMC, adults >50 years; (**D**) The relation between DP and BMD or BMC, adults >50 years; (**E**) The relation between DP and risk of fracture stratified by women or men. SE, standard error.

Three cohort studies [39,52,53] examined a “Prudent” or “Healthy” dietary pattern with fracture (Figure 2E). Among men, a lower risk of fracture was seen for those in the highest versus lowest categories of intake (OR = 0.81; 95% CI: 0.69, 0.95; *p* = 0.01), with no evidence of heterogeneity (*p* = 0.72, *I*^2^ = 0%). Among women, this relationship was not statistically significant (OR = 0.93; 95% CI: 0.78, 1.11; *p* = 0.44), and evidence of heterogeneity was detected (*p* = 0.01, *I*^2^ = 77%).

### 3.6. Western/Unhealthy Dietary Pattern and BMD

Of the nine studies [27,28,29,34,36,42,44,49,50] included in the meta-analysis of “Western/Unhealthy” dietary pattern and BMD, three cross-sectional studies were in children and adolescents [27,28,29], two cross-sectional studies [34,36] were in young adults, and five studies (two cohort [42,44] and three cross-sectional [34,49,50]) were in older adults. Among older adults, a comparison of the highest to the lowest intake category of the “Western/Unhealthy” dietary pattern in cohort studies [42,44] showed a pooled RR for low BMD of 1.53 (95% CI: 1.14, 2.05; *p* = 0.004), with no evidence of heterogeneity (*p* = 0.65, *I*^2^ = 0%). A similar association, which approached significance, was found among cross-sectional studies (34,49,50), with a pooled OR of 1.93 (95% CI: 1.36, 2.75; *p* <0.001), and no evidence of heterogeneity (*p* = 0.59, *I*^2^ = 0%).

#### Western/Unhealthy Dietary Pattern and Risk of Fracture

Three cohort studies [39,52,53] identified an association between a “Western” or an “Unhealthy” dietary pattern and fracture (Figure 2E). Men with intakes in the highest versus lowest categories showed a 10% higher fracture risk (OR = 1.10; 95% CI: 1.02, 1.19; *p* = 0.01), with no evidence of heterogeneity (*p* = 0.90, *I*^2^ = 0%). For women, this association was similar (OR = 1.08; 95% CI: 1.00, 1.17; *p* = 0.06), with no evidence of heterogeneity (*p* = 0.94, *I*^2^ = 0%), but only approached significance.

### 3.7. Risk of Bias in the Studies Included in the Meta-Analysis

Two reviewers (L.M.-S. and E.D.-G.) independently assessed the risk of bias for each study using the criteria outlined in the Cochrane Handbook for Systematic Review of Interventions [55]. Disagreements were solved by discussion or by involving a third author (P.C). We assessed the risk of bias according to the following domains of observational studies: 1. Risk of bias; 2. Inconsistency; 3. Indirectness; 4. Imprecision; and 5. Other considerations (Publication bias, large effect, plausible confounding, and dose–response gradient). We present the risk of bias analysis according to the *GRADEpro* Guideline Development Tool (56) in Table 3.

## 4. Discussion

To our knowledge, this is the first systematic review and meta-analysis of dietary patterns, BMD, and risk of fracture based on “a posteriori” derived dietary patterns. Our meta-analysis suggests that a “Prudent/Healthy” dietary pattern may decrease the risk of low BMD among children and adolescents, young adults, and older adults. Further, our results indicate that a “Western/Unhealthy” dietary pattern may increase the risk of low BMD in adults older than 50 years. Among older men, the pooled results of the cohort studies showed a significant association between “Prudent/Healthy” dietary patterns and a reduced risk of fracture, while “Western/Unhealthy” patterns were related to a higher risk of fracture. Among women, the associations between “Prudent/Healthy” or “Western/Unhealthy” were in the expected direction, but did not achieve statistical significance.

Previous studies have established that relationships between diet (nutrients, foods, or food groups) and low BMD or risk of fracture exist [11,12,13,14,15,16,17,18]. Nutrients, such as calcium, vitamin D, phosphorus, potassium, magnesium, and vitamin K, as well as some food groups (i.e., fruit and vegetables) have shown beneficial effects on bone health and lower risk of fracture [11,12,13,14,15,16,17,18,57,58], while foods such as soft drinks (particularly colas) have been associated with low BMD and a greater risk of fracture [18,59]. Further, “a priori” dietary patterns, such as the Mediterranean diet, which is based on plant foods, such as fruits, vegetables, whole grains, legumes, and nuts, fish, olive oil, and a reduced intake of red meat and saturated fatty acids [60,61], has been positively associated with bone health.

Our analyses identified two consistent “a posteriori” types of dietary patterns: “Prudent/Healthy” and “Western/Unhealthy”. These total dietary patterns may contribute to diverse biological mechanisms that affect BMD and risk of fracture. In general, the “Prudent/Healthy” dietary pattern was characterized by high intakes of fruits, vegetables, whole grains, legumes, nuts, fish, low-fat dairy products, and low-fat milk, and low intakes of soft drinks, sugars, refined grains or cereals, red meat, and processed meat. Previous systematic reviews [62,63,64], have reported that the consumption of fruit and vegetables may reduce the risk of low BMD and fracture. Intake of fruits and vegetables, as well as whole grains, increases intake of several important vitamins, minerals, and phytonutrients, which may contribute to bone health through effects on acid-based balance [65], calcium metabolism [66], antioxidant capacity, which suppresses osteoclast action [67], and bone matrix formation [68], and by decreasing homocysteine concentration [69], among others.

Additional components of the “Prudent/Healthy” dietary pattern include fish and nuts. These contain polyunsaturated fatty acids (particularly *n*-3 fatty acids), which have been associated with anti-inflammatory properties that promote bone health [70]. Finally, dairy products and milk are an important source of calcium, magnesium, vitamin D, and protein, which are needed for bone matrix formation and preservation [71,72].

On the other hand, “Western/Unhealthy” dietary patterns tend to be characterized by red meat, processed meat, soft drinks, refined grains or cereals, fast food, and sweets. These dietary components contribute saturated fats, sodium, added sugars, and phosphorus [73,74], which have been linked to a higher risk of low BMD and fracture incidence. A higher intake of these nutrients contributes to imbalances that may decrease osteoblast differentiation and bone development [75], alter the equilibrium of calcium [76], or contribute to acid load [77].

There are some methodological limitations to the interpretation of our results. First, most of the studies included in our systematic review and meta-analysis were cross-sectional and do not allow for possible changes in diet over time. Second, most of the “a posteriori” dietary patterns included in our analysis were derived through factor analysis methods, which depend on subjective decisions during analysis (definition and number of food groups entered, number of factors to retain) and may lead to variation in pattern definition across studies [19,20,21,78,79,80]. In the studies we reviewed, food groups and loading factors in the dietary patterns derived were not identical between studies. Although this may result in a misclassification bias, the results across studies in the overall dietary patterns identified were quite consistent. To reduce potential bias in the meta-analysis, we selected only those dietary patterns that showed reasonably similar factor loadings for the most frequently consumed food groups. Such a methodology has been employed by other systematic reviews and meta-analyses evaluating dietary patterns and different health outcomes [81,82,83]. Most studies included in the present review and meta-analysis used food frequency questionnaires (FFQs), which often differ across studies. Nonetheless, previous studies comparing dietary patterns derived from FFQs as well as from 24-h dietary recalls or diet histories have found reasonable reproducibility [84,85,86]. Additionally, cultural, racial, and ethnic consumption patterns and food choices differ across populations. Despite this, we found similarities in the foods or food groups included in the different studies, as well as in the dietary pattern definitions. Finally, other dietary patterns could be related to BMD or BMC and fracture risk. In the present study, we only included the most commonly identified dietary patterns (similar foods or food groups); thus, it is possible that other dietary patterns, and, also, dietary patterns derived with different methods, such as an “a priori” score-based approach, could be associated with BMD or BMC and fracture risk.

The studies also varied in the confounding variables used in the analysis. However, most studies adjusted for the main documented risk factors for low BMD and risk of fracture, i.e., sex, age, physical activity, multivitamin use, height, weight, passive or active smoking, and, in women, age at menarche or menopausal status. Still, due to the observational nature of the included studies, residual confounding cannot be ruled out.

The quality of assessed evidence (*GRADEpro* GDT) [56] ranged from “moderate” to “very low”, based on the observational design of the studies included in the meta-analysis. Further issues included identified limitations in study execution (explained withdrawals). In contrast, our study has several strengths, including the lack of identification of serious risk of bias, low heterogeneity across studies (<50%), and clear variability along the scores.

## 5. Conclusions

In summary, the present systematic review and meta-analysis provides evidence of an inverse association between a “Prudent/Healthy” dietary pattern and risk of low BMD across all age groups included. Conversely, a positive relation between “Western/Unhealthy” dietary pattern and low BMD was found only in older adults. Further, the meta-analyses contribute evidence that a “Prudent/Healthy” dietary pattern is protective against fracture risk among men, while a “Western/Unhealthy” dietary pattern is associated with greater fracture incidence. These results should encourage health professionals to emphasize the importance of consuming healthy diets that include fruits, vegetables, whole grains, fish, legumes, nuts, low-fat dairy products, milk, and water, while avoiding refined foods high in saturated fat and added sugars, for the prevention of low BMD and fracture risk.

## Figures and Tables

**Table 1:** The main characteristics of the epidemiological studies on the association between bone mineral density or bone mineral content and dietary patterns defined using the “a posteriori” approach.

Reference	Location	Number of Subjects	Age (years)	Diet-Assessment Method	Dietary Pattern Derivation Method	Pattern Name	Factors Adjusted forin Analyses (Multivariable)	Main Result
**Cohort studies, children and adolescents**								
van den Hooven, et al., 2015 [24]	The Netherlands	2850	6 years	FFQ ^1^	PCA ^2^-factor analysis (varimax rotation)	“Potatoes, rice, and vegetables”, “Refined grains and confectionary”, and “Dairy and whole grains” dietary patterns	Sex, ethnicity, birth weight Z-score, adherence scores for the two-other dietary patterns, total energy intake, time interval between dietary assessment and visit, age at visit, height at visit, weight at visit, and maternal BMI ^3^ at enrolment	Adherence to a “Dairy and whole grains” pattern was positively associated with BMD ^4^. The other patterns were not associated with BMD
Monjardino, et al., 2015 [25]	Portugal	1007	17 years	FFQ	Cluster analysis	“Healthier”, “Dairy products”, “Fast food and sweets”, and “Lower intake” dietary patterns	Height, weight, total energy intake, and age at menarche (in girls)	Among girls, adherence to a “Lower intake” pattern was negatively associated with BMD, compared with subjects with a “Healthier” pattern
Wosje, et al., 2010 [26]	USA ^5^	325	6.8–7.8 years	3-day food records	RRR ^6^	Pattern 1 and pattern 2 (not labeled)	Race, sex, height, weight, energy intake, calcium intake, physical activity, and time spent viewing television and playing outdoors	A pattern characterized by high intakes of dark green vegetables, deep-yellow vegetables, and low intakes of processed meats, fried chicken and fish, and fried potatoes was associated with higher bone mass
**Cross-sectional studies, children and adolescents**								
Mu, et al., 2014 [27]	China	1319	16–20 years	FFQ	Factor analysis (varimax rotation)	Four dietary patterns were identified: “Western food pattern”, “Animal protein pattern”, “Calcium food pattern”, and “Chinese traditional pattern”	Sex, physical activity, economic status, passive smoking, calcium supplements, body mass index	The findings suggested that there was a positive association between a “Chinese traditional” dietary pattern and healthy BMD and that this same association was observed between “Calcium food pattern” and BMD. In contrast, “Western pattern” was negatively related with BMD; however, the relationship was not statistically significant
Shin, et al., 2013 [28]	Korea	196	14.2 years (12–15 years)	6-day Food records	Factor analysis (varimax rotation)	Four different dietary patterns were identified: “Traditional Korean” dietary pattern, “Fast food” dietary pattern, “Milk and cereal” dietary pattern, and “Snacks” dietary pattern	Age, sex, BMI percentiles, weight loss attempts, pubertal status, and exercise	These results indicate that the intake of milk and cereal is important for the bone health of Korean adolescents, whose diets are composed mainly of grains and vegetables
Yang, et al., 2016 [29]	China	1590	11–17 years	FFQ	PCA-factor analysis(varimax rotation)	“Chinese and western”, “Westernization”, and “Meat” dietary patterns	Sex, passive smoking, drinking, calcium supplements, BMI, and physical activity	Rural–urban disparity in dietary patterns was found in this study, and different dietary patterns were associated with the risk of some adverse outcomes
**Cohort studies, young adults and adults**								
van den Hooven, et al., 2015 [30]	Australia	1024	20 years	FFQ	RRR	Pattern 1 and pattern 2 (not labeled)	Sex, ethnicity, age at DXA ^7^, height at DXA, fat mass plus lean mass at DXA, household income, cardiorespiratory fitness, screen time, dietary misreporting, and total energy intake	Subjects with adherence to a pattern 1 (characterized by: High intake of low-fat dairy, whole grains, vegetables, fish, fresh fruits and legumes, and a low intake of refined grains, cakes and cookies, fried potatoes, soft drinks, confectionary, and chips had greater levels of BMD. Subjects with adherence to pattern 2 (represented by: High consumption of red meat, poultry, processed meats, steamed/grilled/canned fish, meat dishes, and eggs; and low intake of dairy products, fresh fruits, fruit juices) had lower levels of BMD
Whittle, et al., 2012 [31]	Northern Ireland	489	20–25 years	7-day diet history	PCA-factor analysis (varimax rotation)	“Healthy”, “Traditional”, “Meats and nuts” only for women, “Refined” only for men, and “Social” dietary patterns	Age, BMI, smoking, physical activity, father’s social class, and energy intake	Women with higher scores of “Meats and nuts” pattern had significantly greater BMD. Men with higher scores of “Refined” pattern had significantly lower BMD. The other patterns were not associated significantly with BMD
McNaughton, et al., 2011 [32]	Australia	525	18–65 years	4-day food diary	PCA-factor analysis (varimax rotation)	Pattern 1, pattern 2, pattern 3, pattern 4, and pattern 5	Age, height, energy intake, smoking, sport, walking, education, calcium intake	A pattern high in processed cereals, soft drinks, fried potatoes, sausages, and processed meats, vegetable oils, beer, and take-away foods was inversely associated with BMD. Subjects with high intake of chocolate, confectionary, added sugar, fruit drinks and cordials, high-fat dairy milk and yoghurt, and unprocessed cereals had lower levels of BMD. A pattern represented by high intakes of leafy vegetables, tomato and tomato products, low-fat dairy milk and yoghurt, fruit, cheese, eggs and egg dishes, fish, sauces, gravies, and salad dressings was associated with higher levels of BMD.
Langsetmo, et al., 2010 [33]	Canada	6539	25–49 years	FFQ	PCA-factor analysis (varimax rotation)	“Nutrient-dense” and “Energy-dense” dietary patterns	Age, height, center, education, smoking, alcohol consumption, activity, sedentary time, milk consumption, supplements (vitamin D, calcium); and antiresorptives, corticosteroids, and recent (<5 years) menopause	The “Nutrient-dense” or “Energy-dense” dietary patterns were not associated significantly with BMD
**Cross-sectional studies, young adults and adults**								
Denova-Gutiérrez, et al., 2016 [34]	Mexico	6915	20–80 years	FFQ	PCA-factor analysis (varimax rotation)	“Prudent”, “Refined foods”, and “Dairy and fish” dietary patterns	Age, gender, BMI, height, multivitamin use, smoking status, physical activity, and energy intake. For women: estrogen use, age of menarche, parity, and menopause	Subjects in the highest quintile of the “Prudent” pattern had lower odds of having low BMD. Subjects in the highest quintile of the “Refined foods” pattern had higher odds of having low BMD. Subjects in the highest quintile of the “Dairy and fish” pattern had lower odds of having low BMD
Mangano, et al., 2015 [35]	USA	2758	29–86 years	FFQ	Cluster analysis	“Chicken”, “Fish”, “Processed foods”, “Red meat”, and “Low-fat milk” dietary patterns	Age, sex, estrogen status, BMI, height, total energy intake, current smoking status, alcohol intake, calcium supplement use and vitamin D supplement use, and physical activity	BMD was higher among subjects in the “Low-fat milk” pattern, compared with subjects in the “Processed foods” and “Red meat” dietary patterns
Shin, et al., 2014 [36]	Korea	1828	46 years	3-day food records	PCA-factor analysis (varimax rotation)	“Rice and Kimchi”, “Eggs, meat, and flour”, “Fruit, milk, and whole grains”, and “Fast food and soda” dietary patterns	Age, body size (weight and height adjusted for weight residual), energy intake, smoking status, alcohol consumption, physical activity, and, for women, menopausal status	Subjects in the highest quartile of the “Fruit, milk, and whole grains” pattern presented lower odds of low BMD. The other patterns were not associated significantly with low BMD
Kontogianni, et al., 2009 [37]	Greece	220	48 years	3-day food records	PCA-factor analysis (varimax rotation)	Pattern 1, pattern 2, pattern 3, pattern 4, pattern 5, pattern 6, pattern 7, pattern 8, pattern 9, and pattern 10 (not labeled)	BMI, smoking status, physical activity level, and low energy reporting	A pattern characterized by high intakes of fish, olive oil, nuts, and vegetables, and low consumption of red meat and products and poultry, was positively associated with BMD
Okubo, et al., 2006 [38]	Japan	291	40–55 years	Diet history questionnaire	PCA-factor analysis (varimax rotation)	“Healthy”, “Japanese traditional”, “Western”, and “Beverages and meats” dietary patterns	Age, BMI, grasping power, current smoking, fracture history, the use of HTR, age at menarche, parity, and use of calcium and multivitamin supplements	Subjects in the highest quintile of the “Healthy” pattern had higher BMD. Subjects in the highest quintile of the “Western” pattern had lower BMD
**Cohort studies, adults ≥50 years**								
de Jonge, et al., 2017 [39]	The Netherland	4028	≥55 years	FFQ	RRR	“Fruit, vegetables, and dairy” and “Sweets, animal fat, and low meat”	Age, sex, body weight, height, vitamin D plasma concentrations, the month of the vitamin D measurement, the use of lipid-lowering drugs, and dietary calcium intake	A “fruit, vegetable, and dairy” pattern was associated with higher BMD ^3^. A “sweets, animal fat, and low meat” pattern was not associated with higher BMD
de Jonge, et al., 2018 [40]	The Netherland	5144	≥55 years	FFQ	PCA (varimax rotation)	“Traditional”, “Health conscious”, and “Processed” dietary patterns	Age, sex, initial body weight and height, total energy intake, and adherence to the other two dietary patterns.	A “Health” dietary pattern may have benefits for BMD. Adherence to a “Processed” pattern may pose a risk for low BMD
Ward, et al., 2016 [41]	United Kingdom	1263	60–64 years	7-day food diary	RRR	Only the first pattern; the “Nutrient-dense” pattern was investigated	Height, weight, social class, geographic region, physical activity, smoking status, supplement use, and time since menopause	A pattern characterized by low fat milk, fruit, low fat yoghurt, vegetables, fish, and fish dishes was associated with higher BMD
Melaku, et al., 2016 [42]	Australia	1182	≥50 years	Dietary questionnaire	PCA (varimax rotation)	“Prudent” and “Western” dietary patterns	Sex, age, socio-economic factors, smoking status, alcohol intake, marital status, income, health literacy, job-related physical activity, diabetes mellitus, a family history of osteoporosis, body mass index, and energy intake	Participants in the highest category of the “Prudent” pattern had a lower prevalence of low BMD. Subjects in the highest category of the “Western” pattern were more likely to have low BMD
Chen, et al., 2015 [43]	China	282	50–65 years	FFQ	PCA (varimax rotation)	“Cereal grains” and “Milk-root vegetables” dietary patterns.	Age, years since menopause, height, weight, systolic blood pressure, waist–hip ratio, change of weight since menopause, age of menophania, educational attainment, occupation, family income, and physical activity level	Subjects with adherence to a “Cereal grains” pattern had lower BMD. Subjects with adherence to a “Milk-root vegetables” pattern had higher hip BMD
Park, et al., 2012 [44]	Korea	1464	≥50 years	FFQ	PCA-factor analysis (varimax rotation)	“Traditional”, “Dairy”, and “Western” dietary patterns	Age, residual area, exercise, and passive smoking	Subjects with adherence to the “Traditional” and “Western” dietary patterns had a higher risk of osteoporosis. Subjects with adherence to a “Dairy” pattern had a lower risk of osteoporosis
Fairweather-Tait, et al., 2011 [45]	United Kingdom	>2000	53 years	FFQ	PCA (varimax rotation)	“Fruit and vegetable”, “High alcohol”, “Traditional English”, “Dieting”, and “Low meat” dietary patterns	Age, age squared, BMI, smoking, and physical activity	Adherence to the “Traditional English” pattern had a negative effect on BMD. No significant associations were observed with the other four dietary patterns.
Pedone, et al., 2011 [46]	Italy	434	65–94 years	FFQ	Cluster analysis	Dietary pattern 1 and Dietary pattern 2 (not labeled)	Age, BMI, physical activity, creatinine clearance	Subjects of dietary pattern 2 were less likely to have a lower BMD compared with subjects in pattern 1
Tucker, et al., 2002 [47]	USA ^6^	907	69–93 years	FFQ	Cluster analysis	“Meat, dairy, and bread”, “Meat and sweet baked products”, “Sweet baked products”, “Alcohol”, “Candy”, and “Fruit, vegetables, and cereal” dietary patterns	BMI, height, age, energy intake, physical activity score, smoking, vitamin D supplement use, calcium supplement use, season, and estrogen use for women	Men and women in the “Candy” pattern had significantly lower BMD than in the “Fruit, vegetables, and cereal” pattern. Men in the “Fruit, vegetables, and cereal” pattern had the greatest average of BMD of all subjects
**Cross-sectional studies, adults ≥50 years**								
De França, et al., 2015 [48]	Brazil	156	68 years	3-day food diary	PCA-factor analysis (varimax rotation)	“Healthy”, “Red meat and refined cereals”, “Low-fat dairy”, “Sweet foods, coffee, and tea”, and “Western” dietary patterns	Energy intake, calcium intake, lean mass, height, and postmenopausal time	The “sweet foods, coffee, and tea” dietary pattern was inversely and significantly associated with BMD. The other patterns were not associated significantly with BMD
Shin, et al., 2013 [49]	Korea	3735	54 years	24-h dietary recall	PCA-factor analysis (varimax rotation)	“Meat, alcohol, and sugar”, “Vegetables and soya sauce”, “White rice, kimchi, and seaweed” and “Dairy and fruit” dietary patterns	Age, BMI, energy intake, parathyroid hormone, serum 25-hydroxyvitamin D, smoking, alcohol intake, moderate physical activity, supplement use, and oral contraceptive use	Subjects in the highest quintile of “White rice, kimchi, and seaweed” pattern had a higher likelihood of osteoporosis Subjects in the highest quintile of “Dairy and fruit” pattern had a lower likelihood of osteoporosis
Karamati, et al., 2012 [50]	Iran	160	50–85 years	FFQ	PCA-factor analysis (varimax rotation)	Dietary pattern 1, Dietary pattern 2, and Dietary pattern 3 (not labeled)	Age, BMI, physical activity, age at menarche, age at menopause, parity, lactation, sunlight exposure, smoking, education, fragility fracture history, history of hormone replacement therapy, supplement intake, and antiresorptive drug use	Subjects in the highest tertile of pattern 1 (high intake of vegetables and fruits, and low intake of nonrefined cereals and refined cereal) had significantly higher BMD compared with those in the lowest tertile
Hardcastle, et al., 2011 [51]	United Kingdom	3236	50–59 years	FFQ	PCA-factor analysis (varimax rotation)	“Healthy”, “Processed foods”, “Bread and butter”, “Fish and chips”, and “Snack food” dietary patterns	Weight, height, current smoking, physical activity level, age, social deprivation category, HRT^8^ use, and menopausal status	Subjects with adherence to the “Processed foods” and “Snack food” dietary patterns had lower BMD. The other patterns were not associated with BMD

^1^ FFQ, Food frequency questionnaire; ^2^ Principal component analysis; ^3^ BMI, body mass index; ^4^ BMD, Bone mineral density; ^5^ USA, United States of America; ^6^ RRR; Reduced Rank Regression; ^7^ DXA, dual-energy X-ray absorptiometry; ^8^ HTR, hormone replacement therapy.

**Table 2:** The main characteristics of the epidemiological studies on the association between risk of fracture and dietary patterns defined using the “a posteriori” approach.

Reference	Location	Number of Subjects	Age (years)	Diet-Assessment Method	Dietary Pattern Derivation Method	Pattern Name	Factors Adjusted for in Analyses (Multivariable)	Main Result
**Cohort studies, adults ≥50 years**								
de Jonge EAL, et al., 2017 [39]	The Netherlands	4028	≥55 years	FFQ ^1^	RRR ^2^	“Fruit, vegetables, and dairy”, “Sweets, animal fat, and low meat”	Age, sex, body weight, height, vitamin D plasma concentrations, the month of the vitamin D measurement, the use of lipid-lowering drugs, and dietary calcium intake	Adherence to the fruit, vegetables, and dairy pattern was associated with a lower risk of fractures (HR ^3^ = 0.92; 95%CI: 0.89, 0.96) and hip fractures (HR = 0.81; 95% CI: 0.70, 0.93). In contrast, adherence to the sweets, animal fat, and low meat pattern was associated with higher hazards of osteoporotic fractures (HR = 1.12; 95%CI: 1.07, 1.16) and hip fractures (HR = 1.14; 95%CI: 1.05, 1.23)
Fung TT, et al., 2015 [52]	USA ^4^	112,845	>50 years	FFQ	PCA ^5^-factor analysis (varimax rotation)	“Prudent” and “Western”	Adjusted for age, physical activity, thiazide use, lasix use, oral anti-inflammatory steroids, body mass index (BMI ^6^), smoking, energy intake, calcium supplement, multivitamin supplement, and postmenopausal hormone use in women. All covariates were time-varying	No significant association was observed with the “Prudent” or “Western” pattern
Langsetmo L, et al., 2011 [53]	Canada	5188	>50 years	FFQ	PCA-factor analysis (varimax rotation)	“Nutrient-dense” and “Energy-dense”	Age, education, cigarette smoking, alcohol, activity, daily milk consumption, daily use of supplements, diagnosis of osteoporosis, history of low-trauma fracture after age 40 years, medication use, and comorbidities	The nutrient-dense dietary pattern was associated with a reduced risk of fracture in women. A similar trend was observed in men. The energy-dense dietary pattern was closer to the null in both women and men
**Case-control studies, adults ≥50 years**								
Zeng F-F, et al., 2013 [54]	China	1162	>55 years	FFQ	PCA-factor analysis (varimax rotation)	“Healthy”, “Prudent”, “Traditional”, “High-fat”	BMI, education, household income, house location, smoking, alcohol consumption, tea drinking, physical activity, daily energy intake, family history of fractures, calcium supplement use, and multivitamin use	Was associated with a 58% (95% CI: 0.27, 0.76) decreased risk of hip fracture for participants whose scores were in the highest tertile for the healthy dietary pattern. The “Prudent” pattern was associated with decreased fracture risk (OR = 0.51; 95%CI: 0.28, 0.90). Individuals in the highest tertile of the “High-fat” pattern had a greater risk of suffering a hip fracture (OR = 2.25; 95%CI: 1.38, 3.69), compared with individuals in the lowest tertile

^1^ FFQ, Food frequency questionnaire; ^2^ RRR; Reduced rank regression; ^3^ HR, hazard ratio; ^4^ USA, United States of America; ^5^ PCA, Principal component analysis; ^6^ BMI, body mass index.

**Table 3:** The risk of bias analysis according to the Cochrane guideline to report the risk of bias analysis (using GRADEpro).

Prudent Dietary Pattern or Western Dietary Pattern for Bone Mineral Density							
Certainty Assessment							Summary of Findings
№ of Participants (Studies) Follow-Up	Risk of Bias	Inconsistency	Indirectness	Imprecision	Publication Bias	Overall Certainty of Evidence	Summary of Findings
**Relation between “Prudent” DP and BMD in Children and adolescents (assessed with: PCA-factor analysis (varimax rotation), Cluster analysis, and RRR)**							
3105 (3 observational studies)	not serious	not serious	not serious	not serious	none	⊕ 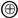 LOW	The dietary patterns: Four dietary patterns were identified: “Western food pattern”, “Animal protein pattern”, “Calcium food pattern”, and “Chinese traditional pattern”. Four different dietary patterns were identified: the “Traditional Korean” dietary pattern, the “Fast food” dietary pattern, “the Milk and cereal” dietary pattern, and the “Snacks” dietary pattern. “Chinese and western”, “Westernization”, and “Meat” dietary patterns
**Relation between “Western” DP and BMD in Children and adolescents (assessed with: Factor analysis (varimax rotation) and PCA-factor analysis (varimax rotation))**							
3105 (3 observational studies)	not serious	not serious	not serious	serious ^a^	none	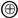 VERY LOW	The dietary patterns: Four dietary patterns were identified: the “Western food pattern”, the “Animal protein pattern”, the “Calcium food pattern”, and the “Chinese traditional pattern”. Four different dietary patterns were identified: the “Traditional Korean” dietary pattern, the “Fast food” dietary pattern, the “Milk and cereal” dietary pattern, and the “Snacks” dietary pattern. “Chinese and western”, “Westernization”, and “Meat” dietary patterns
**Relation between “Prudent” DP and BMD in young adults ≥20 years to <50years (assessed with: PCA-factor analysis (varimax rotation))**							
8743 (2 observational studies)	not serious ^b^	not serious	not serious	not serious	dose response gradient	⊕⊕ 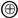 MODERATE	The dietary pattern: Denova-Gutiérrez, et al., 2016: “Prudent”, “Refined foods”, and “Dairy and fish” dietary patterns. Shin, et al., 2014: “Rice and Kimchi”, “Eggs, meat, and flour”, “Fruit, milk, and whole grains”, and “Fast food and soda” dietary patterns
**Relation between “Western” DP and BMD in young adults ≥20 years to <50years (assessed with: PCA-factor analysis (varimax rotation))**							
8743 (2 observational studies)	not serious	not serious	not serious	serious ^c^	dose response gradient ^b^	⊕ 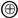 LOW	The dietary pattern: Denova-Gutiérrez, et al., 2016: “Prudent”, “Refined foods”, and “Dairy and fish” dietary patterns. Shin, et al., 2014: “Rice and Kimchi”, “Eggs, meat, and flour”, “Fruit, milk, and whole grains”, and “Fast food and soda” dietary patterns
**Relation between “Prudent” DP and BMD in adults ≥50years (follow up: mean 2 years; assessed with: PCA (varimax rotation); PCA-factor analysis (varimax rotation); or Cluster analysis)**							
3080 (3 observational studies)	not serious	not serious	not serious	not serious	dose response gradient ^d^	⊕⊕ 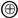 MODERATE	The dietary pattern: “Prudent” and “Western” dietary patterns. The “Traditional”, “Dairy”, and “Western” dietary patterns. Dietary pattern 1 and Dietary pattern 2 (not labeled)
**Relation between “Western” DP and BMD in adults ≥50years (follow up: mean 2 years; assessed with: PCA (varimax rotation); PCA-factor analysis (varimax rotation); or Cluster analysis)**							
3080 (3 observational studies)	not serious	not serious	not serious	not serious	strong association	⊕⊕ 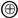 MODERATE	The dietary pattern: “Prudent” and “Western” dietary patterns. The “Traditional”, “Dairy”, and “Western” dietary patterns. Dietary pattern 1 and Dietary pattern 2 (not labeled)
**Relation between “Prudent” DP and BMD in adults ≥50years (assessed with: PCA-factor analysis (varimax rotation))**							
3895 (2 observational studies)	not serious	not serious	not serious	not serious	dose response gradient ^e^	⊕⊕ 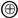 MODERATE	The dietary pattern: “Meat, alcohol, and sugar”, “Vegetables and soya sauce”, “White rice, kimchi, and seaweed” and “Dairy and fruit” dietary patterns. Dietary pattern 1, Dietary pattern 2, and Dietary pattern 3 (not labeled).
**Relation between “Western” DP and BMD in adults ≥50years (assessed with: PCA-factor analysis (varimax rotation))**							
3895 (2 observational studies)	not serious	not serious	not serious	serious ^f^	dose response gradient ^e^	⊕ 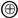 LOW	The dietary pattern: “Meat, alcohol, and sugar”, “Vegetables and soya sauce”, “White rice, kimchi, and seaweed” and “Dairy and fruit” dietary patterns. Dietary pattern 1, Dietary pattern 2, and Dietary pattern 3 (not labeled)
**Relation between “Prudent” DP and risk of fracture in WOMEN (assessed with: RRR: Reduced rank regression; PCA-factor analysis (varimax rotation))**							
122,061 (3 observational studies)	not serious	serious ^g^	not serious	serious ^h^	none	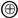 VERY LOW	The dietary patterns: Dietary pattern 1 (“Fruit, vegetables, and dairy”, “Sweets, animal fat, and low meat”); Dietary pattern 2 (“Prudent or western”); and Dietary pattern 3 (“Nutrient-dense” and “Energy-dense”).
**Relation between “Western” DP and risk of fracture in WOMEN (assessed with: RRR: Reduced rank regression; PCA-factor analysis (varimax rotation))**							
122,061 (3 observational studies)	not serious	not serious	not serious	not serious	none	⊕ 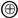 LOW	The dietary patterns: Dietary pattern 1 (“Fruit, vegetables, and dairy”, “Sweets, animal fat, and low meat”); Dietary pattern 2 (“Prudent or western”); and Dietary pattern 3 (“Nutrient-dense” and “Energy-dense”).
**Relation between “Prudent” DP and risk of fracture in MEN (assessed with: RRR: Reduced rank regression; PCA-factor analysis (varimax rotation))**							
122,061 (3 observational studies)	not serious	not serious	not serious	not serious	none	⊕ 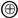 LOW	The dietary patterns: Dietary pattern 1 (“Fruit, vegetables, and dairy”, “Sweets, animal fat, and low meat”); Dietary pattern 2 (“Prudent or western”); and Dietary pattern 3 (“Nutrient-dense” and “Energy-dense”).
**Relation between “Western” DP and risk of fracture in MEN (assessed with: RRR: Reduced rank regression; PCA-factor analysis (varimax rotation))**							
122,061 (3 observational studies)	not serious	not serious	not serious	not serious	none	⊕ 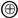 LOW	The dietary patterns: Dietary pattern 1 (“Fruit, vegetables, and dairy”, “Sweets, animal fat, and low meat”); Dietary pattern 2 (“Prudent or western”); and Dietary pattern 3 (“Nutrient-dense” and “Energy-dense”).

CI: Confidence interval. Explanations: ^a^ The Western DP value odds ratio (OR) = 1.09 (95%IC 0.82–1.44); ^b^ In the article by Denova, et al., the analysis was done by categorizing the score in quintiles, and there is a clear gradient along the scores, which even in the document are significant. Additionally, in the article by Shin, et al., the analysis is carried out with the score divided into quartiles and a gradient is also observed along said quartiles, which are significant; ^c^ The Western DP value OR = 1.30 (95%IC 0.76–2.24); ^d^ In the article by Melaku, et al., the scores are divided into tertiles. There is a gradient along the tertiles and the values are significant. In the article by Park, et al., the scores are divided into quintiles. There is a gradient along the quintiles and the values are significant; ^e^ In the cases of Karamati et al. (score divided into tertiles) and Shin et al. (score divided into quintiles), a response gradient is observed along the pattern. It was not always significant; ^f^ The Western DP value OR = 1.79 (95%IC 0.98–3.28); ^g^ The Heterogeneity value I2 = 77%; ^h^ The Prudent DP value OR = 0.93 (95%IC 0.78–1.11).
